# Salicylic acid in plant immunity and beyond

**DOI:** 10.1093/plcell/koad329

**Published:** 2024-01-02

**Authors:** Steven H Spoel, Xinnian Dong

**Affiliations:** Institute of Molecular Plant Sciences, School of Biological Sciences, University of Edinburgh, The King's Buildings, Edinburgh EH9 3BF, UK; Department of Biology, Howard Hughes Medical Institute, Duke University, Durham, NC 27708, USA

## Abstract

As the most widely used herbal medicine in human history and a major defence hormone in plants against a broad spectrum of pathogens and abiotic stresses, salicylic acid (SA) has attracted major research interest. With applications of modern technologies over the past 30 years, studies of the effects of SA on plant growth, development, and defence have revealed many new research frontiers and continue to deliver surprises. In this review, we provide an update on recent advances in our understanding of SA metabolism, perception, and signal transduction mechanisms in plant immunity. An overarching theme emerges that SA executes its many functions through intricate regulation at multiple steps: SA biosynthesis is regulated both locally and systemically, while its perception occurs through multiple cellular targets, including metabolic enzymes, redox regulators, transcription cofactors, and, most recently, an RNA-binding protein. Moreover, SA orchestrates a complex series of post-translational modifications of downstream signaling components and promotes the formation of biomolecular condensates that function as cellular signalling hubs. SA also impacts wider cellular functions through crosstalk with other plant hormones. Looking into the future, we propose new areas for exploration of SA functions, which will undoubtedly uncover more surprises for many years to come.

## Salicylic acid (SA): a multifaceted plant hormone with a deep history in human civilizations

For over 4,000 years, humanity has been familiar with the plant hormone SA and its derivatives. As documented in ancient inscriptions and texts, willow (Latin name *Salix*) and myrtle, which contain substantial amounts of SA derivatives, were used as herbal medicines by ancient Assyrians, Egyptians, and Chinese civilizations, while Hippocrates was said to have made use of willow bark extracts for reducing fever and pain. Similarly, SA-rich meadowsweet was a sacred medicinal herb utilized by Druids of the ancient Celts. However, it was not until the early 19th century that SA and its derivative, salicin, were isolated from willow bark by Johann Andreas Buchner, Henri Leroux, and Raffaele Piria, and from meadowsweet by Löwig and Weidmann, respectively (Norn et al. 2009). This eventually led to the discovery of SA-derived aspirin as a major innovation in modern medicine for the alleviation of pain and prevention of cardiovascular disease and even cancer (Norn et al. 2009).

A large body of evidence from the last 50 years demonstrates that plants also utilize SA as a multifaceted endogenous agent for healing plant ailments. It plays important roles in mitigating abiotic stresses, including heat, cold, drought, UV radiation, heavy metals, and osmotic shock (Rivas-San Vicente and Plasencia 2011). Moreover, SA is both a direct and indirect regulator of plant development, influencing processes such as seed germination, growth, photosynthesis, thermogenesis, flowering, and senescence (Rivas-San Vicente and Plasencia 2011). But SA is best known for its role in orchestrating plant immune responses.

Plant immune systems are composed of several layers of sophisticated mechanisms. Conserved microbe-associated molecular patterns are detected by cell surface-localized pattern recognition receptors (PRRs) that activate pattern-triggered immunity (PTI). PTI induces diverse cell signaling events, including the production of reactive oxygen species (ROS) and biosynthesis of SA, as well as other defense hormones (Couto and Zipfel 2016). Collectively, these signaling events lead to induction of immune genes to confer resistance. However, adapted pathogens promote their virulence by suppressing PTI responses through secretion of effectors directly into the host. To negate this, plants have evolved intracellular nucleotide-binding domain leucine-rich repeat (NLR) immune receptors that detect the presence of pathogen effectors and launch effector-triggered immunity (ETI) (Jones et al. 2016). Immune pathways activated by pattern recognition receptors and NLRs display mutual potentiation. Consequently, simultaneous detection of the pathogen through both receptors leads to stronger immune responses that are often associated with programmed cell death (PCD) of the infected tissues, thereby isolating and killing the invading pathogen (Ngou et al. 2021, 2022; Yuan et al. 2021). Such responses can induce SA synthesis in both local and systemic tissues, where SA may have contrasting roles as a cell death agonist and as a cell survival signal to confer long-lasting protection throughout the plant against a wide variety of pathogens (Rate and Greenberg 2001; Torres et al. 2005; Lu et al. 2009; Fu et al. 2012; Zavaliev et al. 2020). This inducible immune mechanism, characterized by A. Frank Ross as systemic acquired resistance (SAR) in 1961 (Ross 1961), has since been extensively studied due to its potential as an immunizing strategy to protect crops against a broad spectrum of pathogens (White 1979; Ward et al. 1991; Friedrich et al. 1996; Gorlach et al. 1996; Lawton et al. 1996; Shimono et al. 2012; Fu and Dong 2013; Vlot et al. 2021). Surprisingly, although SA is synthesized locally upon infection and detected throughout the plant (Malamy et al. 1990; Metraux et al. 1990; Gaffney et al. 1993), grafting experiments demonstrated that de novo synthesis of SA in systemic tissues is required for the establishment of SAR (Gaffney et al. 1993; Vernooij et al. 1994).

In the past 30 years, significant progress has been made in the understanding of SA biosynthesis in both local and systemic tissues, SA perception by metabolic enzymes and receptor proteins, SA-induced biomolecular condensates in different plant tissues and subcellular compartments, and SA crosstalk with other hormones and metabolites. As a complementary update to previously published reviews (Ding and Ding 2020; Lefevere et al. 2020) and in celebration of ASPB's 100th birthday, we focus on more recent breakthroughs in our understanding of the functions of SA in plants.

## Precise regulation of local and systemic SA biosynthesis

Although basal SA levels differ between organs and plant species, pathogen-induced biosynthesis of SA is an almost universal step toward the establishment of immunity. Plants have evolved 2 major pathways for SA biosynthesis catalyzed by PHENYLALANINE AMMONIA-LYASE (PAL) and ISOCHORISMATE SYNTHASE (ICS), respectively. Different species utilize these pathways to varying degrees. For example, whereas the PAL pathway is more prevalent in rice, the ICS pathway is predominantly used in *Arabidopsis* (Lefevere et al. 2020; Wu et al. 2023). Both pathways utilize chorismate produced in chloroplasts, which in case of the PAL pathway is exported to the cytoplasm, where it is converted to phenylalanine in a multistep process. PAL then converts phenylalanine to trans-cinnamic acid from which SA is generated through the sequential action of ABNORMAL INFLORESCENCE MERISTEM 1 (AIM1) and a yet-to-be-identified benzoic acid hydrolase. In rice, mutating *PAL4* and *PAL6* reduce SA accumulation and enhance susceptibility to various pathogens, indicating these 2 genes play a predominant role in pathogen-induced SA biosynthesis (Duan et al. 2014; Tonnessen et al. 2015). By contrast, *Arabidopsis* encodes for 2 ICS enzymes, with ICS1 being responsible for the accumulation of SA in leaf tissues in response to biotic or abiotic stress (Wildermuth et al. 2001; Garcion et al. 2008). Recent findings revealed that the ICS1-catalyzed conversion of chorismate to isochorismate is the only step of the SA biosynthesis pathway that is localized in the chloroplasts (Rekhter et al. 2019). Isochorismate is then transported from the chloroplast to the cytosol by the MATE transporter protein ENHANCED DISEASE SUSCEPTIBILITY 5 (EDS5), mutants of which show severely decreased SA accumulation upon pathogen infection (Nawrath and Métraux 1999; Nawrath et al. 2002). In the cytosol, isochorismate is conjugated to glutamate by the amidotransferase avrPphB SUSCEPTIBLE 3 (PBS3), where the resulting product, isochorismate-9-glutamate, is converted to SA either by the acyltransferase ENHANCED PSEUDOMONAS SUSCEPTIBILITY 1 or by spontaneous decomposition (Rekhter et al. 2019; Torrens-Spence et al. 2019).

Although these advances revealed most steps in the SA biosynthesis pathways of plants, relatively little is known about how the enzymes in these pathways are regulated. Reports show that PAL isozymes are ubiquitinated by a SKP1-CULLIN1-F-BOX (SCF)-type E3 ligase and targeted for proteasome-mediated degradation, suggesting post-translational regulation of enzyme activities could be a key regulatory step in SA biosynthesis (Zhang et al. 2013, 2015).

In contrast to the regulation of enzymatic activities, transcriptional regulation of SA synthesis genes has been extensively studied and demonstrated as a major control point in the biosynthesis of SA. The 2 most-studied regulators of SA biosynthesis genes are the related transcriptional activators SAR DEFICIENT 1 (SARD1) and CALMODULIN BINDING PROTEIN 60 g (CBP60g). Mutants of these transcription factors (TFs) fail to induce SA synthesis upon infection and are deficient in both local and systemic immune responses (Wang et al. 2009; Zhang et al. 2010). These TFs were found to bind to the *ICS1* promoter and also associate with the promoters of *EDS5* and *PBS3*, suggesting that they have a regulatory role in the entire SA biosynthesis pathway (Wang et al. 2011; Truman and Glazebrook 2012; Sun et al. 2015). SARD1 and CBP60g also have functions beyond SA biosynthesis because they were found to bind promoters of key signaling proteins downstream of SA, as well as promoters of positive and negative regulators of PTI, ETI, and SAR (Sun et al. 2015). Curiously, another related member of the CBP60 family, CBP60b, was shown to control the expression of *SARD1*, suggesting that CBP60 family members also fine-tune each other's expression levels (Huang et al. 2021b). The biological relevance of the CBP60 family is perhaps best illustrated by the fact that the vascular pathogen *Verticillium dahlia* secretes an effector that directly inhibits CBP60g transcriptional activity to promote its virulence in both *Arabidopsis* and cotton plants (Qin et al. 2018). In the plant pathogen arms race, however, it is thought that members of the CBP60 family have influenced each other's evolution to generate a robust immune regulatory module that is more resilient to perturbation by pathogen effectors (Zheng et al. 2022). Because transcription of both *SARD1* and *CBP60g* genes are induced upon pathogen infection, it raises the question: how are these activators induced upon pathogen challenge? One possible mechanism is activation of CBP60g by PTI-induced calcium transients, because CBP60g's calmodulin-binding domain is required for accumulation of SA and pathogen resistance (Wang et al. 2009). Moreover, calcium transients may also regulate the activities of CALMODULIN-BINDING TRANSCRIPTION ACTIVATOR (CAMTA) TFs that suppress SA biosynthesis via direct repression of *CBP60g* and *SARD1* gene expression (O’Malley et al. 2016; Kim et al. 2020; Sun et al. 2020).

The answer to the question above may also lie with other TFs, including WRKYs, TEOSINTE BRANCHED1/CYCLOIDEA/PCF (TCP) family TFs, NO APICAL MERISTEM ARABIDOPSIS TRANSCRIPTION ACTIVATION FACTOR AND CUP-SHAPED COTYLEDON (NAC) family TFs, DP-E2F-LIKE1 (DEL1), ethylene-responsive ETHYLENE INSENSITIVE3 (EIN3), and ETHYLENE INSENSITIVE3-LIKE1 (EIL1) TFs, all of which have been shown to activate or repress the expression of SA biosynthesis genes (Chen et al. 2009; van Verk et al. 2011; Zheng et al. 2012, 2015; Wang et al. 2015). Of particular interest is NTM1-LIKE 9, a potentially membrane-bound TF that, upon activation, may be cleaved and released to induce *ICS1* gene expression specifically in guard cells, where SA biosynthesis is required for stomatal closure in response to pathogen attack (Zheng et al. 2015). Moreover, TCP21, also known as CHE (CCA1 HIKING EXPEDITION), is a circadian clock TF that regulates daily rhythms of basal SA biosynthesis by binding to the *ICS1* promoter (Zheng et al. 2015). Curiously, CHE is required for pathogen-induced SA biosynthesis only in systemic tissues during the establishment of SAR. CHE contains a conserved, redox-sensitive cysteine residue in its noncanonical basic helix-loop-helix DNA-binding domain (Viola et al. 2013). Upon local induction, H_2_O_2_ derived from NADPH oxidases, such as RESPIRATORY BURST OXIDASE HOMOLOG PROTEIN D (RBOHD), functions as a mobile signal to induce *S*-sulfenylation (-SOH group) of this cysteine residue in systemic tissues, which enhances CHE's binding affinity for the *ICS1* promoter and stimulates SA synthesis (Cao et al. 2023). CHE is also modified in local tissues, but here, higher H_2_O_2_ levels lead to further oxidation of the cysteine residue to the *S*-sulfinylated state (-SO_2_H group) and possibly even to the *S*-sulfonated state (-SO_3_H group), neither of which appears to induce CHE's binding affinity to the promoter of *ICS1*. Thus, a gradient of mobile H_2_O_2_ combined with concentration-dependent sensing by CHE regulates spatial SA biosynthesis in the establishment of SAR. It is tempting to hypothesize that plants modulate whether and how strongly to turn on SAR based on the severity of the local infection as reflected by the H_2_O_2_ levels. Although the discovery of the H_2_O_2_-CHE signaling pathway addressed the 30-year-old question of how a local infection leads to de novo SA synthesis in systemic tissues during SAR, the mechanism by which pathogens and other environmental stresses initiate local SA synthesis remains to be fully elucidated.

## SA is perceived directly by multiple target proteins to influence distinct cellular processes

How is SA, a simple phenolic molecule, perceived in the cell to establish immunity? The profound impacts of SA on plant and human physiology suggest that it is likely to have many target proteins. Indeed, screens utilizing SA analogs in combination with protein arrays or crosslinking revealed nearly 100 SA-binding proteins (SABPs) (Tian et al. 2012; Moreau et al. 2013; Manohar et al. 2015). Several of these have already been confirmed to play a role in SA signaling and plant defense, supporting the notion that there is unlikely a single receptor for SA or a single paradigm for SA perception, but rather many ways by which SA directly alters cellular responses through binding to metabolic enzymes, redox regulators, and transcription cofactors.

Metabolic enzymes that bind SA include the *β*-carbonic anhydrase SABP3, the acyl acid amido synthetase GH3, the amidotransferase PBS3 described above, glyceraldehyde 3-phosphate dehydrogenases, alpha-ketoglutarate dehydrogenase, and thimet oligopeptidases (TOP1 and TOP2) (Pokotylo et al. 2019). SA binding to these enzymes often influences their activities, suggesting SA can reprogram cellular metabolism. However, the biological consequences of such interactions have yet to be demonstrated. Therefore, these SABPs may be important gateways toward understanding the role of SA in metabolic reprogramming that favors immune responses over other cellular activities.

SA also targets redox regulators. Treatment with SA or SA analogs has been shown to trigger rapid cellular oxidation followed by reduction as measured by changes in reduced vs oxidized glutathione levels (Mou et al. 2003; Spoel and Loake 2011). Consistent with this observation, the earliest identified cellular target of SA was the ROS-scavenging enzyme CATALASE 2 (CAT2). Binding of SA reduces the H_2_O_2_-detoxifying activity of CAT2, allowing H_2_O_2_ to accumulate upon pathogen infection and function as a secondary messenger in SAR (Chen et al. 1993; Conrath et al. 1995; Wang et al. 2014; Cao et al. 2023). Moreover, it was shown that SA-mediated suppression of CAT2 activity leads to inhibitory *S*-sulfenylation of TRYPTOPHAN SYNTHETASE B SUBUNIT 1 (TSB1) involved in auxin biosynthesis, and additionally, CAT2 inhibition may also limit jasmonic acid (JA) biosynthesis (Yuan et al. 2017). Thus, redox regulation through direct SA binding is likely to coordinate the plant hormone signaling network. Besides CAT2, SA also inhibits the enzymatic activities of GST enzymes in vitro (Tian et al. 2012). Although the biological relevance of this remains unknown, GST enzymes are involved in the production of anti-microbial compounds, cellular detoxification, and hormone transport (Gullner et al. 2018), suggesting the possible influence of SA on these processes. Further, SA binds to the chloroplast-localized THIOREDOXIN (TRX) *m*1 (Manohar et al. 2015). Although the effect of SA on this enzyme remains unknown, the TRX family of oxidoreductases play key roles in immunity by fine-tuning cellular redox homeostasis, enabling oxidative signaling, and protecting proteins from the damaging effects of hyper-oxidation (Mata-Pérez and Spoel 2019; Bleau and Spoel 2021).

Besides enzymes, SA binds to members of the NONEXPRESSOR OF *PR* GENE (NPR) family of transcriptional cofactors (Fu et al. 2012; Manohar et al. 2015; Ding et al. 2018; Wang et al. 2020; Kumar et al. 2022). NPR1 is arguably the most important cellular target/receptor of SA, as illustrated by its repeated identification in genetic screens as an essential component for SA-mediated gene expression and resistance (Cao et al. 1994, 1997; Delaney et al. 1995; Glazebrook et al. 1996; Ryals et al. 1997; Shah et al. 1997). NPR1 acts as a major coactivator that establishes a transcriptional activation complex consisting of NPR1, TGA TFs, and histone acetyltransferases (Fan and Dong 2002; Jin et al. 2018). NPR1 associates with numerous gene promoters to reprogram the expression of thousands of genes (Wang et al. 2005, 2006; Skelly et al. 2019; Nomoto et al. 2021). Although nuclear translocation of NPR1 is induced through SA-triggered redox changes (Mou et al. 2003; Tada et al. 2008), whether NPR1's transcriptional coactivator activity requires direct association with SA has been hotly debated (Fu et al. 2012; Manohar et al. 2015; Ding et al. 2018; Wang et al. 2020; Kumar et al. 2022), because NPR1 exhibited much lower SA-binding activity in side-by-side comparisons with its paralogs, NPR3 and NPR4 (Fu et al. 2012; Wang et al. 2020). The crystal structure of the NPR4 SA-binding core (SBC) revealed that SA is located in an enclosed hydrophobic pocket made of 4 α-helices, indicating that SA may induce a conformational change to accommodate its binding to NPR4 (Wang et al. 2020). Although NPR1 has nearly identical hormone-binding residues as NPR4, nonconserved residues in the SBC appear to be responsible for its low SA-binding activity (Wang et al. 2020). Recent cryo-EM and crystal structure analyses demonstrate that the active form of full-length NPR1 is a bird-shaped homodimer with interacting N-terminal BROAD-COMPLEX, TRAMTRACK AND BRIC-À-BRAC (BTB) domains forming the “body” of the bird and each extending “wing” consisting of a Kelch helix bundle, 4 ankyrin repeats (ANK) and a disordered C-terminal domain (Kumar et al. 2022). The presence of SA induces folding of the C-terminal SA-binding domain (SBD) in vitro and promotes its docking onto the ANK repeat domain. Crosslinking of this docked conformation by artificially engineering a disulfide bond at the interface resulted in enhanced activation of the NPR1 target gene, *PR1*. This finding provides the first structural evidence for a direct role of SA in inducing conformational changes in NPR1 to promote its transcriptional activity. However, it remains to be elucidated why docking of the SA-bound SBD onto ANKs helps enhance TGA activity because there is no direct contact between the ANK-docked SBD and TGA (Kumar et al. 2022). A possible explanation is that a post-translational modification or an unknown chaperone might be required for enhancing in vivo SA binding to NPR1 and bridging the docked SBD with TGA, thus bringing the transcriptional machinery to the TFs.

**Figure 1.**
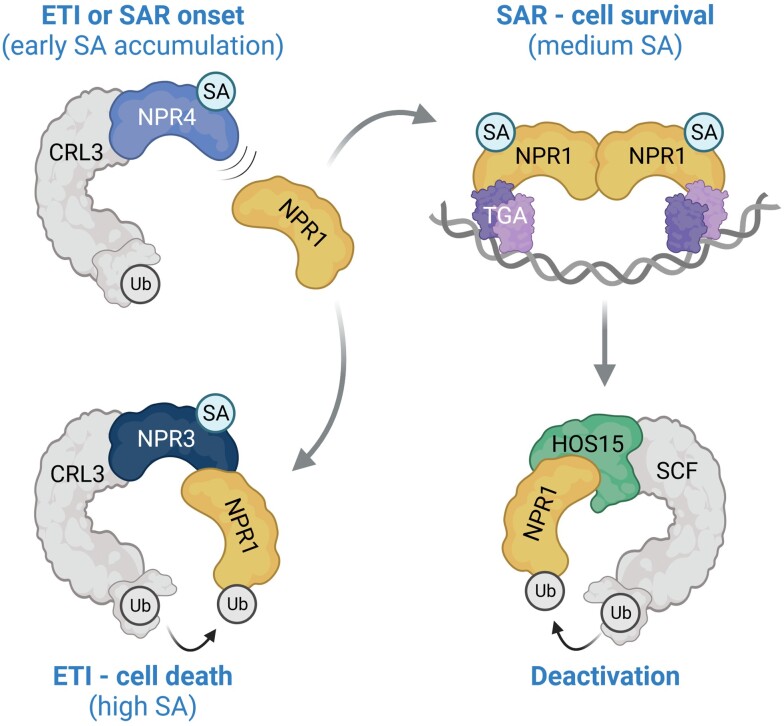
NPR1 is regulated by multiple E3 ubiquitin ligases. Under steady-state conditions, NPR1 is targeted for degradation by a CRL3^NPR4^ ubiquitin ligase to avoid untimely activation of immunity. At the early onset of immunity, SA begins to accumulate and binds to NPR4, which prevents this protein from interacting with NPR1 (top left). This allows NPR1 to activate gene expression and promote cell survival during SAR (top right). Activation of ETI leads to much higher levels of SA, which promote recruitment of NPR1 to a CRL3^NPR3^ ubiquitin ligase that targets NPR1 for degradation, thereby permitting cell death to occur (bottom left). Alternatively, the transcriptionally competent state of NPR1 in SAR can be deactivated by the SCF^HOS15^ ubiquitin ligase (bottom right). Created with BioRender.com.

In contrast to NPR1, NPR3 and NPR4 proteins are negative regulators of SAR and exhibit significantly higher SA binding activities in vitro (Zhang et al. 2006; Fu et al. 2012). SA binding to NPR3 and NPR4 controls their interactions with NPR1 in opposite ways: whereas SA disrupts the NPR4-NPR1 complex, it promotes interaction between NPR3 and NPR1. The SA dependency of these interactions suggests that the C-terminal half of the protein, containing the ANK-repeat domain and the SBD domain, is likely involved (Fu et al. 2012; Wu et al. 2012; Wang et al. 2020). What are the functional consequences of these unusual SA-dependent interactions between NPR3/4 and NPR1 proteins? The domain structures and organization of NPR proteins are typical for substrate adaptors utilized by CULLIN 3-RING LIGASE (CRL3). These substrate adaptors typically contain a BTB domain that interacts with CRL3, whereas an additional protein-protein interaction domain (e.g. the ANK-repeat domain in NPR proteins) recruits the substrate for (poly)ubiquitination, which in many cases leads to substrate degradation by the proteasome (Petroski and Deshaies 2005). Indeed, in the nucleus, NPR3 and NPR4 were shown to function as adaptors for CRL3 to target NPR1 and JASMONATE ZIM DOMAIN (JAZ) corepressors of the JA signaling pathway for degradation in an SA-regulated manner (Fu et al. 2012; Liu et al. 2016). Because SA levels taper off with distance from the site of infection, NPR3/NPR4 provide a way to spatially regulate the level of NPR1: at the site of infection, NPR1 is ubiquitinated and degraded to remove its inhibitory effect on cell death, whereas in adjacent and more distal tissues, NPR1 accumulates to promote cell survival and SAR (Fig. 1) (Fu et al. 2012; Liu et al. 2016). This is a unique mode of action in which one CRL3 substrate adaptor targets another for ubiquitination and degradation in a ligand concentration-dependent fashion to ensure appropriate regulation of cell fate upon pathogen challenge.

Alternative to their CRL3 substrate adaptor function, NPR3 and NPR4 have also been proposed to function as transcriptional corepressors. The C termini of NPR3 and NPR4 contain an ETHYLENE-RESPONSIVE ELEMENT BINDING FACTOR-ASSOCIATED AMPHIPATHIC REPRESSION (EAR) motif widely found in transcriptional (co)repressors. Sequence alignment showed that the EAR motif is uniquely absent in NPR1 proteins of *Brassicaceae* but not NPR1s of other plant lineages (Wang et al. 2020). Moreover, SA binding to NPR4 does not appear to affect the EAR motif conformation or the interaction of NPR3 and NPR4 with TGA TFs (Ding et al. 2018; Wang et al. 2020). Therefore, how SA alleviates the proposed NPR4 transcription corepressor activity remains to be investigated.

## SA-mediated post-translational regulation of NPR1 activities

**Figure 2.**
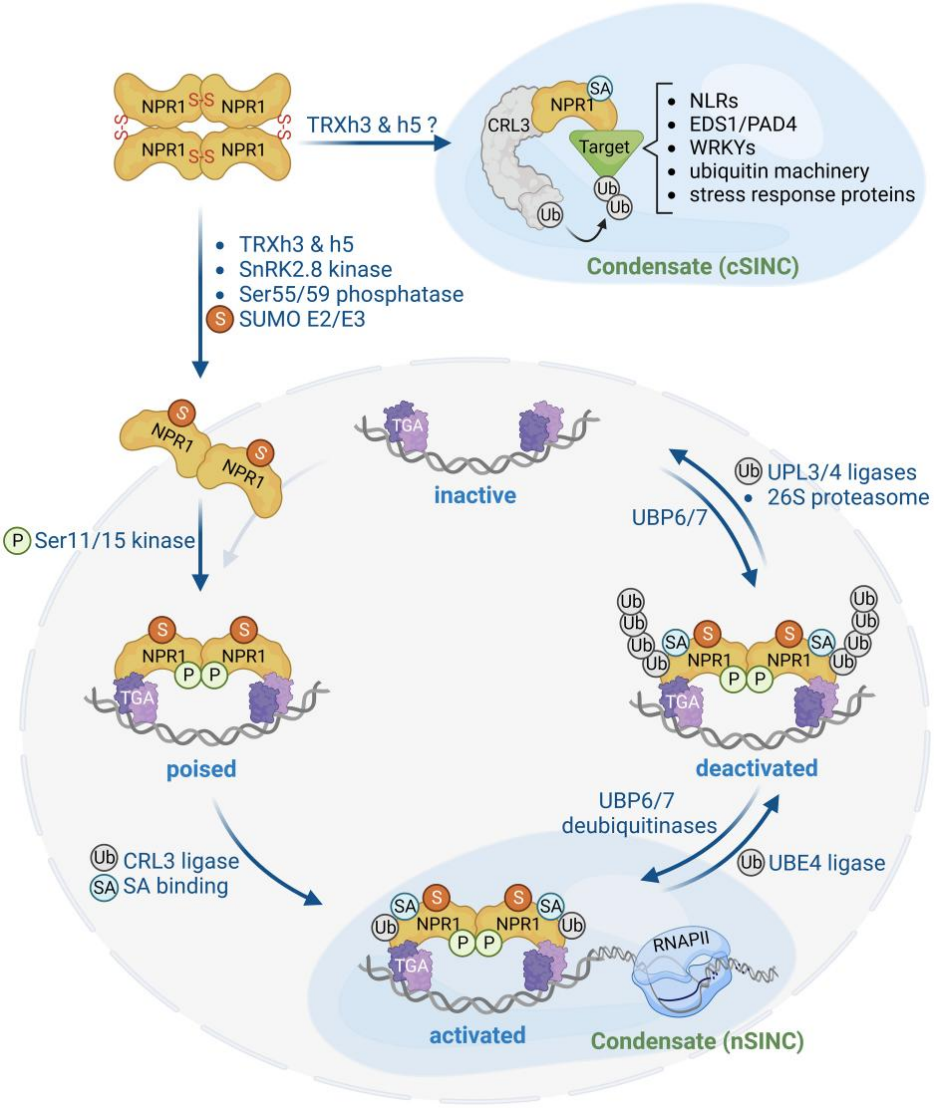
Diverse PTMs dynamically regulate NPR1 localization and activities. In resting cells, NPR1 resides in the cytoplasm as a disulfide-linked (S-S) oligomer. Activation of immunity leads to the TRX*h*3- and TRX*h*5-mediated reduction of NPR1 oligomers and nuclear translocation of NPR1 promoted by SnRK2.8-mediated phosphorylation. Moreover, dephosphorylation of NPR1 at Ser55/59 and its SUMOylation both stimulate localization of NPR1 to the nuclear condensate (nSINC). SUMOylation also promotes NPR1's association with TGA transcription factors and is a prerequisite for phosphorylation (P) at Ser11/15, which stimulates NPR1's transcriptional activity by recruiting a CRL3 ligase that (mono)ubiquitinates NPR1. Sumo (S) and/or ubiquitin (Ub) may act as a molecular chaperone for SA binding, leading to a transcriptionally active NPR1-TGA complex. Eventually, ubiquitin chain elongation by an UBE4 ligase inactivates NPR1 and targets it to the proteasome. At the proteasome UPL3/4 ligases further decorate NPR1 with ubiquitin, which prevents its stalling during degradation and promotes proteasome processivity. NPR1 can be rescued from degradation and returned to its transcriptionally active state by the activities of proteasome-associated UBP6/7 deubiquitinases. In addition to its nuclear function, high SA levels, as found in tissues surrounding ETI-induced cell death, lead NPR1 to form cytoplasmic condensates (cSINCs), where it serves as a CRL3^NPR1^ ligase that targets various cell death-inducing immune regulators for degradation to promote cell survival. Created with BioRender.com.

The multifaceted functions of SA are reflected not only in its various cellular targets, but also in the complex post-translational modifications (PTMs) of its downstream signaling component NPR1 (Fig. 2). Although NPR1 is localized to both the cytoplasm and the nucleus, its conformation is different in these cellular compartments. In the cytoplasm, the majority of NPR1 are linked through intermolecular disulfide bonds to form a homo-oligomer (Mou et al. 2003). Mutational and subsequent structural analyses revealed that at least 2 cysteines are involved in generating this oligomer: Cys82 faces its counterpart at the dimer interface, whereas 2 surface-exposed Cys156 residues of the dimer are juxtaposed at the interface of the tetramer (Kumar et al. 2022). Importantly, the solvent exposed Cys156 can be *S*-nitrosylated (i.e. covalent attachment of nitric oxide), which facilitates further oxidation to a disulfide bond (Tada et al. 2008). SA-induced cellular reduction along with the action of the oxidoreductases TRX*h*3 and TRX*h*5 reduce the NPR1 oligomer, promoting its nuclear translocation (Fig. 2).

Nuclear translocation of NPR1 also requires its phosphorylation at Ser589 and possibly Thr373 by the kinase SnRK2.8 (SNF1-Related Protein Kinase 2.8) and dephosphorylation at Ser55/59 by an unknown phosphatase (Lee et al. 2015; Saleh et al. 2015). In the nucleus, NPR1 undergoes a series of additional PTMs that alter its coactivator behavior (Fig. 2). First, dephosphorylation of Ser55/59 promotes its interaction with the ubiquitin-like modifier, SUMO3, via a SUMO-interaction motif. The resulting SUMOylated NPR1 preferentially associates with TGA3 TF to be recruited to the chromatin (Saleh et al. 2015). Moreover, SUMOylation of NPR1 is a prerequisite for subsequent phosphorylation of a Ser11/15-containing phosphodegron (Saleh et al. 2015), which recruits a CRL3 ligase to ubiquitinate NPR1 (Spoel et al. 2009). Intriguingly, ubiquitination and subsequent degradation of NPR1 were found to enhance its transcription cofactor activity instead of inhibiting it (Spoel et al. 2009). This conundrum is resolved by the discovery of an intricate ubiquitination relay on NPR1 (Fig. 2). First, CRL3 decorates NPR1 with multi-monoubiquitin or short ubiquitin chains that promote its association with target promoters and enhance its coactivator activity without triggering degradation (Skelly et al. 2019). Therefore, ubiquitination and/or SUMOylation of NPR1 are both possible intramolecular chaperones that may induce conformational changes in NPR1 to facilitate SA binding to the SBD pocket. To test this hypothesis, the site(s) of these PTMs in NPR1 need to be identified. Moreover, after serving its transcriptional coactivator function, NPR1 is further ubiquitinated by the E4 ligase UBE4/MUSE3, which deactivates NPR1 and renders it a substrate for the proteasome (Skelly et al. 2019). Upon arrival at the proteasome, the proteasome-associated HECT-type ligases UPL3 and UPL4 further ubiquitinate NPR1, which promotes proteasome processivity by preventing stalling during its degradation (Wang et al. 2022; Wang and Spoel 2022).

Importantly, cells have several “go/no-go” decision points along the ubiquitin ligase relay that decide the fate of NPR1. First, an SCF ligase containing the F-box protein HIGH EXPRESSION OF OSMOTICALLY RESPONSIVE GENES 15 (HOS15) may counteract CRL3-mediated activation of NPR1 by preferentially associating with the Ser11/15 phosphorylated isoform and targeting it directly to the proteasome without activating gene expression (Fig. 2) (Shen et al. 2020). Second, the proteasome-associated deubiquitinases UBP6 and UBP7 reverse ubiquitination by trimming NPR1 ubiquitin chains en bloc, thereby returning NPR1 back to its transcriptionally active state (Fig. 2) (Skelly et al. 2019). Taken together, these findings illustrate how SA utilizes a variety of PTMs to dynamically regulate NPR1 coactivator activity (Fig. 2). Nonetheless, further work is required to understand how SA controls activities of the enzymes that “write” these PTMs.

## SA signaling via biomolecular condensates

The large number of cellular targets and profound physiological impacts of SA on plant cells call for centralized organization and dynamic regulatory mechanisms. Such mechanisms have presented themselves to us under the microscope as SA-induced NPR1-GFP nuclear condensates (nSINCs) (Saleh et al. 2015). However, interest in the potential functional importance of these biomolecular condensates was only raised after the serendipitous discovery that higher concentrations of SA also trigger the formation of SA-induced NPR1 condensates in the cytoplasm (cSINCs) (Zavaliev et al. 2020). These cSINCs, which are unique to NPR1 among the NPR paralogs, contain components of the ubiquitin machinery and numerous stress-responsive proteins, including 10 NLR intracellular immune receptors and their downstream signaling components, ENHANCED DISEASE SUSCEPTIBILITY 1 (EDS1) and PHYTOALEXIN-DEFICIENT 4 (PAD4), required for ETI-associated PCD, as well as redox enzymes and DNA damage response proteins. Transition of NPR1 into cSINCs is required for the formation of a CRL3^NPR1^ ligase complex that targets at least some of these cSINC-containing proteins for ubiquitin-mediated degradation (Fig. 2). cSINCs form only in tissues adjacent to ETI-induced cell death zones where SA concentrations are high. Thus, it is hypothesized that the cSINC-localized CRL3^NPR1^ ligase plays an essential role in promoting cell survival in tissues neighboring ETI-induced cell death by sequestering/degrading the PCD-promoting immune receptors and signaling components.

SA was also found to induce guanylate-binding protein-like GTPases (GBPLs) that assemble condensates in the nucleus. Upon immune activation, catalytically active GBPL3 was found to translocate into the nucleus, where it localizes to the nuclear pore complex and initiates the formation of GBPL defense-activated condensates (GDACs) (Huang et al. 2021a; Tang et al. 2022). GDACs sequester major immune gene promoters, including those of *ICS1*, *EDS5*, *CBP60g*, *SARD1*, and *NPR1*, to a local environment enriched in transcriptional coactivators of the Mediator complex and the RNA polymerase II machinery (Huang et al. 2021a; Kim et al. 2022). Because GBPL3 does not contain any transcriptional activation domains, it appears to act as an SA-induced nucleator to recruit the transcription machinery for reprogramming the transcriptome in response to pathogen threat. It is plausible that the SA-induced NPR1 condensates observed in the nucleus serve a similar hub function as GBPL3 condensates, but instead of regulating SA biosynthesis genes, nSINCs may target downstream SA-responsive genes. Moreover, a recent report demonstrated that upon infection by virulent and avirulent pathogens, MOS4-associated complex (MAC) components form nuclear condensates proposed to activate immunity by sequestering negative regulators of plant defense (Jia et al. 2023). Whether SA plays a role in this process requires further investigation.

In addition to the regulation of defense transcription and protein homeostasis, SA and other phenolic acids have been shown to induce the formation of stress granules in root cells through direct interaction with RNA-BINDING PROTEIN 47B (RBP47B) to inhibit global translation, a mechanism used by plants to suppress growth of their neighboring plants (Xie et al. 2023). Collectively, these findings indicate that SA can orchestrate distinct immune responses by organizing corresponding cellular machineries in close quarters to increase reaction efficiencies and kinetics, as reported for other biological processes in eukaryotic organisms (Banani et al. 2017). The ability of SA-induced NPR1 to form both nuclear and cytoplasmic condensates with distinct components and biological functions provides a promising new research direction to explore the conditions and regulatory mechanisms of biomolecular condensates. So far, mutating the *redox-sensitive intrinsically disordered region 3* (*rdr3*) in NPR1 abolished both nSINC and cSINC formation as well as SA-mediated gene expression and resistance (Zavaliev et al. 2020).

## Synergistic and antagonistic crosstalk between SA and other cellular signals

It is well-known that the establishment of SAR requires de novo SA synthesis in systemic tissue (Gaffney et al. 1993; Vernooij et al. 1994). Recent discovery of H_2_O_2_ as a mobile signal for activation of CHE in systemic tissue to induce SA synthesis (Cao et al. 2023) raises questions about the individual functions of this signal and previously reported systemic signals, including N-hydroxy-pipecolic acid (NHP) (Hartmann et al. 2018), azelaic acid (Jung et al. 2009), glycerol-3-phosphate (Chanda et al. 2011), nitric oxide (Wang et al. 2014), dehydroabietinal (Chaturvedi et al. 2012), monoterpenes (Riedlmeier et al. 2017; Wenig et al. 2019), trans-acting small interfering RNAs (tasi-RNAs) (Shine et al. 2022), and extracellular nicotinamide adenine dinucleotide (phosphate) [eNAD(P)] (Wang et al. 2019; Li et al. 2023) and their possible relationships with SA in inducing SAR. So far, there is strong evidence for synergistic interactions between SA and NHP. Pathogen-induced SA accumulation in local tissues first triggers NHP synthesis, which then feedback amplifies SA synthesis in systemic tissues. Moreover, both signals are inactivated by the same glycosyltransferase (Bauer et al. 2021; Cai et al. 2021; Holmes et al. 2021; Mohnike et al. 2021). Recent work demonstrates that NPR1 is required for NHP-induced SAR and associated transcriptional reprogramming (Yildiz et al. 2021). Similarly, genetic analyses suggest that NPR1-interacting TGA TFs are also required for NHP-induced SAR and SAR-related gene expression (Yildiz et al. 2023). Thus, NHP appears to utilize the SA-responsive NPR1/TGA regulatory module to establish SAR. The striking structural similarity between SA and NHP suggests that NPR1 might be a receptor not only for SA but also for NHP. However, isothermal titration calorimetry assays indicated that NHP was unable to bind to recombinant NPR1 derived from insect cells (Nair et al. 2021). Whether the SA and NHP signals converge on NPR1 or further downstream on NPR1-dependent target genes involving NHP-responsive transcriptional regulators remains to be discovered. Moreover, it has yet to be investigated if, like SA, NHP utilizes similar post-translational strategies to regulate NPR1 coactivator activity.

**Figure 3.**
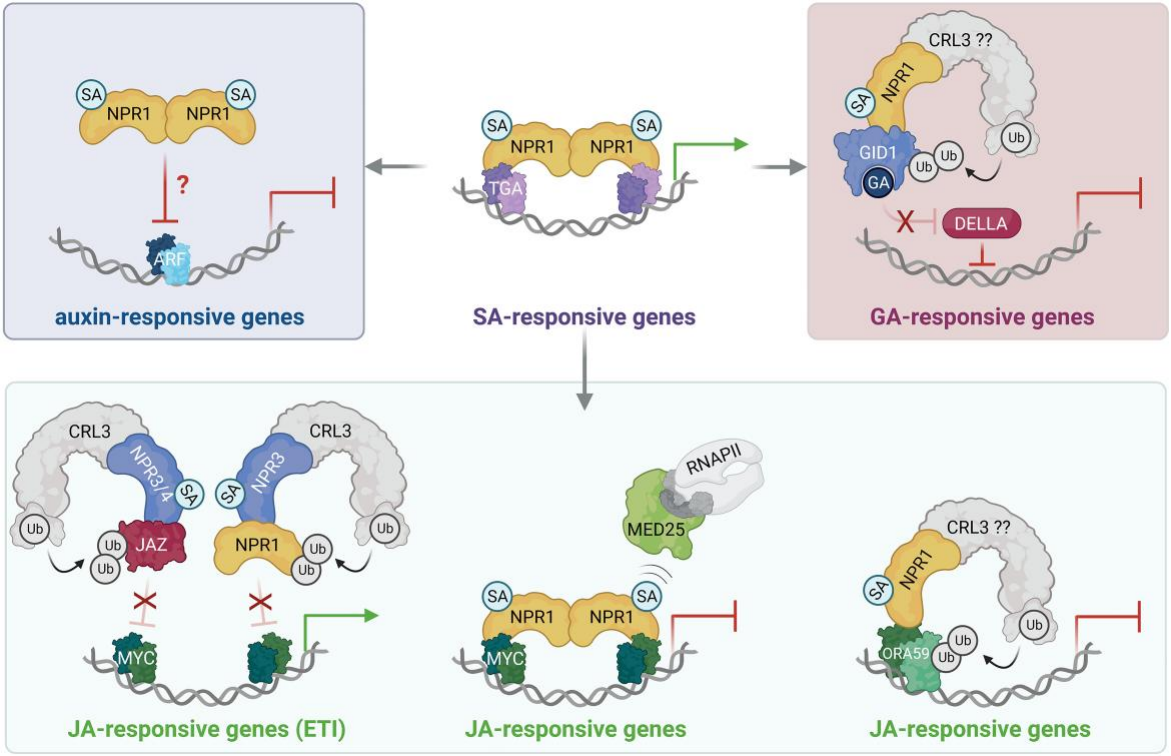
NPR proteins mediate crosstalk between SA and other hormones. While NPR1 is essential for activation of SA-responsive genes during SAR (top center), it can also function as a potent inhibitor of JA-, GA-, and possibly auxin-responsive gene expression. NPR1 inhibits JA-responsive gene expression either by degrading ORA59 activators as part of a CRL3^NPR1^ ligase (bottom right) or by blocking MYC activators' access to Mediator components (MED25) and associated RNA Polymerase II (RNAPII) complex (bottom center). In contrast to NPR1, both NPR3 and NPR4 activate JA-responsive genes during ETI by serving as a CRL3^NPR3/4^ ligase to degrade the JA repressors JAZ and NPR1 (bottom left). Auxin-responsive genes are also inhibited by SA, but whether this process is dependent on NPR1 remains unknown (top left). Lastly, a probable CRL3^NPR1^ ligase inhibits GA signaling by targeting the GA receptor GID1 for degradation, which blocks the removal of DELLA suppressors from GA-responsive genes (top right). Created with BioRender.com.

Reciprocal antagonism between the SA and JA signals has been widely reported and plays an important role in shaping the outcome of plant-pathogen interactions (Pieterse et al. 2009). For example, strains of the biotrophic leaf pathogen *Pseudomonas syringae* promote their virulence by utilizing the JA-mimicking toxin coronatine that functions as a suppressor of SA-mediated immune responses against this pathogen (Brooks et al. 2005; Zheng et al. 2012; Geng et al. 2014). Some plant hosts have turned the tables by developing strategies to block the virulence-promoting activity of coronatine. For example, *Arabidopsis* employs SA signaling itself to antagonize coronatine- or JA-induced responses, with SA-induced redox changes and glutathione biosynthesis playing a key role in the suppression of JA signaling (Koornneef et al. 2008; Pieterse et al. 2009). While SA suppresses the expression of several JA biosynthesis genes, SA also antagonizes signaling downstream of JA biosynthesis, which is dependent on NPR1 (Spoel et al. 2003; Leon-Reyes et al. 2010). SA-induced NPR1 likely utilizes different mechanisms to suppress JA-responsive genes. First, SA and NPR1 induce the expression of GRX480, a member of the glutaredoxin family that interacts with TGA TFs. This GRX480/TGA complex may associate with a subset of JA-responsive genes and suppress their expression (Ndamukong et al. 2007). Second, SA suppresses genes that contain the JA-responsive GCC-box motif, which is bound by members of the APETALA2/ETHYLENE RESPONSE FACTOR (AP2/ERF) TF family. SA was reported to inhibit JA-responsive accumulation of the AP2/ERF transcriptional activator ORA59, indicating that SA antagonizes JA signaling downstream of the nuclear JA receptor complex, SCF^COI1^-JAZ (Van der Does et al. 2013). It is therefore plausible that an SA-induced CRL3^NPR1/3/4^ ubiquitin ligase targets ORA59 for proteasome-mediated degradation (Fig. 3). Lastly, SA-induced NPR1 was recently shown to be recruited to G-box motifs highly overrepresented in JA-responsive promoters, where it associates with MYC transcriptional activators (Nomoto et al. 2021). Like JAZ corepressors, NPR1 interacts with the same N-terminal region of MYC activators and competitively prevents recruitment of the Mediator complex, thereby blocking JA-mediated activation of gene expression. Thus, NPR1 is a versatile SA-responsive transcriptional cofactor that can be deployed either as a coactivator or as a corepressor depending on transcriptional context (Fig. 3).

The outcome of crosstalk between the SA and JA signals is modulated by a number of different factors, including their concentrations, spatial distributions, temporal effects, and even the presence of other hormones. For example, the presence of ethylene renders antagonisms between SA and JA signaling independent of NPR1 (Leon-Reyes et al. 2009). Because ethylene is produced during specific plant-pathogen interactions and abiotic stress responses, plant cells may use ethylene to direct NPR1 activity to specific immune pathways. Additionally, spatial regulation of crosstalk ensures that SA and JA are only antagonistic at the site of infection and not in distal systemic tissues (Spoel et al. 2007). This ensures plants can defend themselves against simultaneous attacks by multiple pathogens with different lifestyles. Spatial regulation may be conferred by gradients of hormone concentrations, which determine if SA and JA interactions are synergistic, antagonistic, or absent (Mur et al. 2006). On the other hand, temporal regulation of basal SA and JA levels by the circadian clock to peak at dawn and dusk, respectively, also indicates that plants have evolved mechanisms to avoid the antagonistic effects of these 2 defense hormones (Goodspeed et al. 2013). In fact, during ETI, high concentrations of SA and JA accumulate without apparent antagonism, because in this scenario JA synthesis/responses are not initiated through the canonical SCF^COI1^ ubiquitin ligase, but rather through the SA receptors NPR3 and NPR4 (Liu et al. 2016). These SA receptors were found to target JAZ corepressors for proteasome-mediated degradation, presumably through their ability to form CRL3^NPR3/4^ ubiquitin ligases, which was required for immune receptor-mediated ETI (Fig. 3) (Fu et al. 2012). These findings suggest that SA not only induces ETI and associated local defense responses but also boosts JA responses to prevent vulnerability to necrotrophic pathogens and/or insects.

Hormones other than JA are also antagonized by SA. Many pathogens synthesize auxin-like molecules or alter the host's auxin homeostasis to enhance their virulence (Spoel and Dong 2008). It has been shown that SA strongly inhibits auxin signaling by preventing the degradation of auxin-related transcriptional corepressors, thereby limiting the activation of auxin-responsive gene expression (Wang et al. 2007). The exact molecular mechanisms by which SA inhibits auxin signaling are unknown, but it is plausible that NPR1 mediates this antagonism. Regardless, this crosstalk plays an important role in suppressing pathogen virulence. In addition, NPR1 was also found to suppress gibberellin (GA) signaling by interacting with the GA receptor GA INSENSITIVE DWARF 1 (GID1), possibly as part of a CRL3^NPR1^ ubiquitin ligase that targets GID1 for proteasome-mediated degradation (Yu et al. 2022). NPR1-mediated degradation of GID1 enhances the stability of downstream DELLA transcriptional corepressors, thereby suppressing GA-responsive gene expression and associated plant growth responses (Fig. 3).

Collectively, these reports on crosstalk between SA and other hormones begin to paint a picture in which the NPR family of SA receptors play a central role. Their dual function as transcriptional cofactors and as substrate adaptors for CRL3 ubiquitin ligases alter the activities or stabilities of other transcriptional activators and corepressors, thereby extending their reach far beyond the regulation of only SA responses into the realms of other plant hormones (Fig. 3).

## Future outlook

As described in this review, the profound impacts of SA on plant and animal physiology, as revealed through years of studies, match its functional complexity and sophisticated regulatory mechanisms. However, there is still a lot more to learn about this small molecule that is full of wonders. Some major questions that need to be addressed include the following: (1) How is SA synthesis initiated in local tissue upon different pathogen and abiotic challenges? It is equally plausible that a common mechanism is used in response to all stimuli or distinct mechanisms are triggered by different stimuli; (2) How does SA exert its distinct functions spatially and temporally? Methods with higher resolutions, such as single-cell sequencing (Nobori et al. 2023; Zhu et al. 2023) and high-affinity biosensors (Chen et al. 2019; Yang et al. 2020), are now becoming available and may soon provide detailed answers; (3) What is the PTM and the associated enzyme or chaperone that helps SA bind to NPR1 to activate the defense transcriptome? (4) How does SA regulate the activities of enzymes that decorate NPR1 and possibly other NPR receptors with PTMs? (5) How does SA control the formation of different biomolecular condensates with distinct biological functions in various plant tissues and subcellular compartments? (6) How is SA perceived in plant species such as rice where the role of NPR proteins is less pronounced? Instead, rice utilizes the transcriptional activator WRKY45 to activate immune gene expression (Shimono et al. 2007; Nakayama et al. 2013). Similar to *Arabidopsis* NPR1, the transcriptional activity of rice WRKY45 is also regulated by phosphorylation and the nuclear ubiquitin-mediated proteasome (Matsushita et al. 2013; Ueno et al. 2015, 2017; Adams and Spoel 2018). Future genetic and biochemical screens may reveal how rice and other species perceive pathogen-induced SA accumulation and transduce signals. By addressing all these questions, we will be able to better understand the multifaceted functions of SA to improve plant and human lives.

## Data Availability

No new data were generated or analysed in support of this work.

## Dive Curated Terms

The following phenotypic, genotypic, and functional terms are of significance to the work described in this paper:

NPR4 Gramene: AT4G19660
NPR4 Araport: AT4G19660

NPR3 Gramene: AT5G45110
NPR3 Araport: AT5G45110

CAT2 Gramene: AT4G35090
CAT2 Araport: AT4G35090

EDS5 Gramene: AT4G39030
EDS5 Araport: AT4G39030

ORA59 Gramene: AT1G06160
ORA59 Araport: AT1G06160

WRKY45 Gramene: AT3G01970
WRKY45 Araport: AT3G01970

ANK Gramene: AT5G54610
ANK Araport: AT5G54610

CCA1 Gramene: AT2G46830
CCA1 Araport: AT2G46830

GRX480 Gramene: AT1G28480
GRX480 Araport: AT1G28480

SUMO3 Gramene: AT5G55170
SUMO3 Araport: AT5G55170

NPR1 Gramene: At1g64280
NPR1 Araport: At1g64280

TGA3 Gramene: AT1G22070
TGA3 Araport: AT1G22070

CBP60g Gramene: AT5G26920
CBP60g Araport: AT5G26920

ETI Gramene: Effector-triggered immunity
ETI Araport: Effector-triggered immunity

PTI Gramene: pattern-triggered immunity
PTI Araport: pattern-triggered immunity

GBPL3 Gramene: AT5G46070
GBPL3 Araport: AT5G46070

COI1 Gramene: AT2G39940
COI1 Araport: AT2G39940

HOS15 Gramene: At5g67320
HOS15 Araport: At5g67320

CUL3 Gramene: AT1G26830
CUL3 Araport: AT1G26830
